# 93. Early Recognition and Response to Increases in Surgical Site Infections (SSI) using Optimized Statistical Process Control (SPC) Charts – the Early 2RIS Trial: A Multicenter Stepped Wedge Cluster Randomized Controlled Trial (RCT)

**DOI:** 10.1093/ofid/ofab466.093

**Published:** 2021-12-04

**Authors:** Arthur W Baker, Iulian Ilieş, James C Benneyan, Yuliya Lokhnygina, Katherine R Foy, Sarah S Lewis, Brittain A Wood, Esther Baker, Linda Crane, Kathryn L Crawford, Andrea Cromer, Polly W Padgette, Linda Roach, Linda Adcock, Nicole Nehls, Joseph Salem, Dale W Bratzler, Patch Dellinger, Linda R Greene, Susan S Huang, Christopher Mantyh, Deverick J Anderson

**Affiliations:** 1 Duke University School of Medicine, Durham, North Carolina; 2 Northeastern University, Boston, MA; 3 Duke University, Durham, NC; 4 Duke Infection Control Outreach Network (DICON), Morrisville, North Carolina; 5 Duke Infection Control Outreach Network, Durham, North Carolina; 6 Oklahoma University Health Sciences Center, Oklahoma City, OK; 7 University of Washington School of Medicine, Seattle, Washington; 8 University of Rochester Medical Center Affiliate, Rochester, New York; 9 University of California, Irvine, Irvine, CA; 10 Duke Center for Antimicrobial Stewardship and Infection Prevention, Durham, North Carolina

## Abstract

**Background:**

Traditional approaches for SSI surveillance have deficiencies that can delay detection of SSI outbreaks and other clinically important increases in SSI rates. Optimized SPC methods for SSI surveillance have not been prospectively evaluated.

**Methods:**

We conducted a prospective multicenter stepped wedge cluster RCT to evaluate the performance of SSI surveillance and feedback performed with optimized SPC plus traditional surveillance methods compared to traditional surveillance alone. We divided 13 common surgical procedures into 6 clusters (Table 1). A cluster of procedures at a single hospital was the unit of randomization and analysis, and 105 total clusters across 29 community hospitals were randomized to 12 groups of 8-10 clusters (Figure 1). After a 12-month baseline observation period (3/2016-2/2017), the SPC surveillance intervention was serially implemented according to stepped wedge assignment over a 36-month intervention period (3/2017-2/2020) until all 12 groups of clusters had received the intervention. The primary outcome was the overall SSI prevalence rate (PR=SSIs/100 procedures), evaluated with a GEE model with Poisson distribution.

Table 1

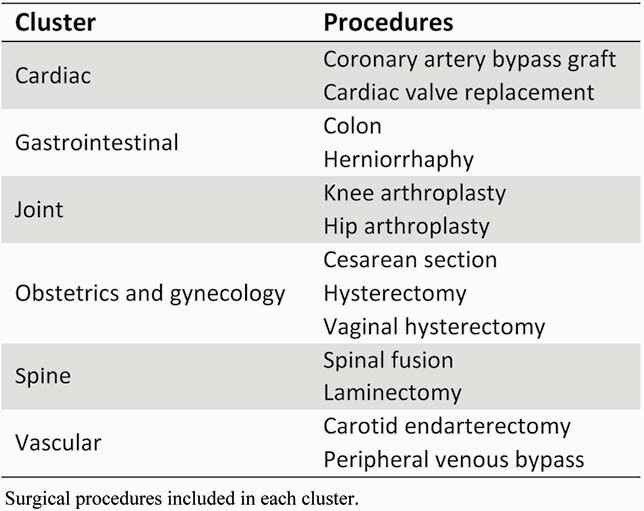

Figure 1

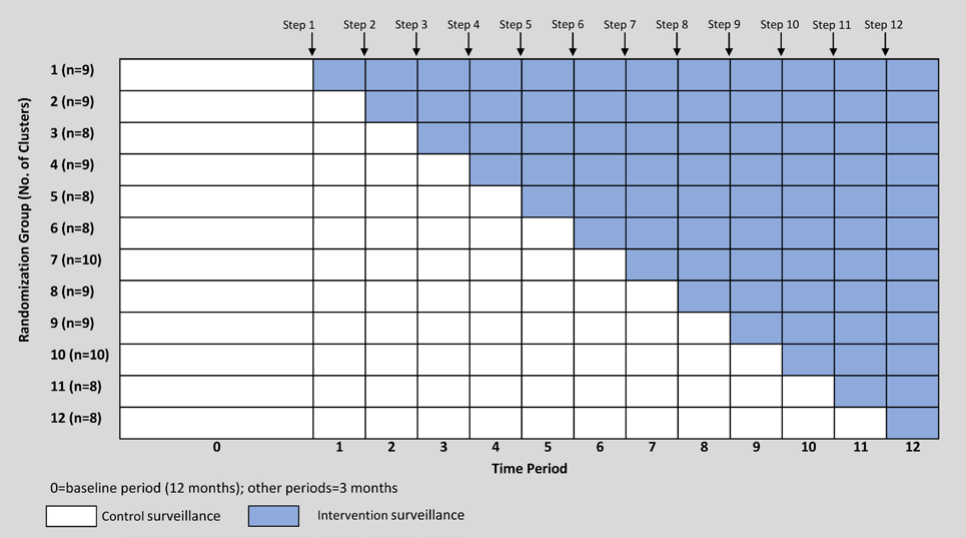

Schematic for stepped wedge design. The 12-month baseline observation period was followed by the 36-month intervention period, comprised of 12 3-month steps.

**Results:**

Our trial involved prospective surveillance of 237,704 procedures that resulted in 1,952 SSIs (PR=0.82). The overall SSI PR did not differ significantly between clusters of procedures assigned to SPC surveillance (781 SSIs/89,339 procedures; PR=0.87) and those assigned to traditional surveillance (1,171 SSIs/148,365 procedures; PR=0.79; PR ratio=1.10 [95% CI, 0.94–1.30]; P=.25) (Table 2). SPC surveillance identified 104 SSI rate increases that required formal investigations, compared to only 25 investigations generated by traditional surveillance. Among 10 best practices for SSI prevention, 453 of 502 (90%) SSIs analyzed due to SPC detection of SSI rate increases had at least 2 deficiencies (Table 3).

Table 2

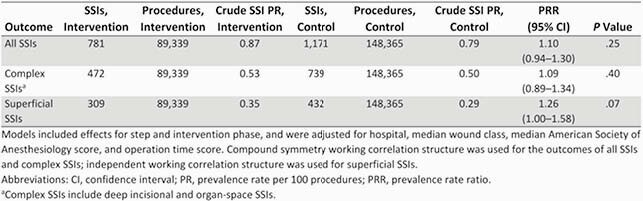

Poisson regression models comparing surgical site infection (SSI) prevalence rates for procedure clusters receiving statistical process control surveillance to SSI rates for clusters receiving traditional control surveillance.

Table 3

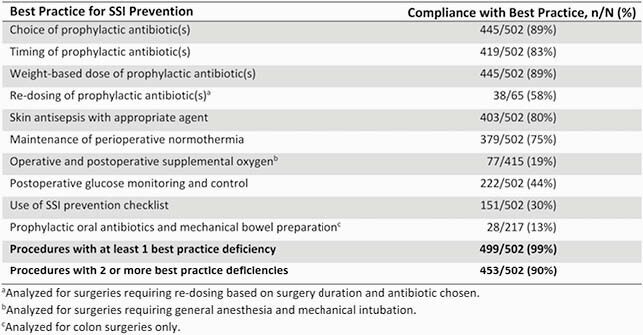

Compliance with 10 best practices for surgical site infection (SSI) prevention among 502 SSIs analyzed during SSI investigations generated by statistical process control surveillance.

**Conclusion:**

SPC methods more frequently detected important SSI rate increases associated with deficiencies in SSI prevention best practices than traditional surveillance; however, feedback of this information did not lead to SSI rate reductions. Further study is indicated to determine the best application of SPC methods to improve adherence to SSI quality measures and prevent SSIs.

**Disclosures:**

**Arthur W. Baker, MD, MPH**, **Medincell** (Advisor or Review Panel member) **Susan S. Huang, MD, MPH**, **Medline** (Other Financial or Material Support, Conducted studies in which participating hospitals and nursing homes received contributed antiseptic and cleaning products)**Molnlycke** (Other Financial or Material Support, Conducted studies in which participating hospitals and nursing homes received contributed antiseptic and cleaning products)**Stryker (Sage**) (Other Financial or Material Support, Conducted studies in which participating hospitals and nursing homes received contributed antiseptic and cleaning products)**Xttrium** (Other Financial or Material Support, Conducted studies in which participating hospitals and nursing homes received contributed antiseptic and cleaning products)

